# Stretching and relaxing the plantar fascia may change plantar fascia thickness but not pressure pain thresholds: a cross-sectional study of patients with plantar fasciopathy

**DOI:** 10.1186/s12891-020-03833-x

**Published:** 2020-12-03

**Authors:** Stefanie Ostermann, Jens Lykkegaard Olesen, Sinéad Holden, Henrik Riel

**Affiliations:** Center for General Practice at Aalborg University, Fyrkildevej 7, 9220 Aalborg East, Denmark

**Keywords:** Plantar fasciopathy, Ultrasonography, Pressure pain threshold, Joint positioning

## Abstract

**Background:**

Despite the established relevance of ultrasonography and assessment of pressure pain thresholds in patients with plantar fasciopathy, patient and probe positioning has been mostly ignored and are not necessarily reported in research. The primary aim of this study was to compare plantar fascia thickness in stretched and relaxed positions in patients with plantar fasciopathy. The secondary aim was to compare plantar heel pressure pain thresholds in these positions.

**Methods:**

In this cross-sectional study, we measured the plantar fascia thickness with ultrasonography, and localised pressure pain thresholds using pressure algometry of 20 patients with plantar fasciopathy. These were assessed bilaterally, with the plantar fascia in both a stretched and relaxed position. In the stretched position, toes were maximally dorsiflexed, while in the relaxed position participants’ feet were hanging freely over the end of the table.

**Results:**

The plantar fascia of the most symptomatic foot was significantly thicker when stretched compared with the relaxed position (sagittal: mean difference 0.2 mm, 95%CI: 0.1–0.4, *P* = 0.013; frontal: mean difference − 0.27, 95%CI: − 0.49 to − 0.06, *P* = 0.014). The plantar fascia was significantly thinner in the frontal plane compared with the sagittal plane in both positions (stretched: mean difference − 0.2 mm, 95%CI: − 0.42 to − 0.03, *P* = 0.025; relaxed: mean difference − 0.3 mm, 95%CI:-0.49 to − 0.08, *P* = 0.008). There was no difference between pressure pain thresholds in stretched or relaxed positions in either foot (*P* > 0.4).

**Conclusions:**

The plantar fascia was significantly thicker in a stretched compared with a relaxed position and in the sagittal compared with the frontal plane, but differences were smaller than the standard deviation. Pressure pain thresholds were not different between the positions. These results highlight the importance of how ultrasonography is performed and reported in research to allow for replication.

**Trial registration:**

The study was pre-registered September 25th, 2017 on ClinicalTrials.gov (NCT03291665).

## Background

Pain at the calcaneal attachment of the plantar fascia, referred to as plantar fasciopathy, is a common cause of chronic heel pain and is prevalent in both athletic and general populations [1–3]. Patients often complain of a pain when getting out of bed in the morning that improves with ambulation [4]. Despite evidence-based treatments such as foot orthoses [5, 6], shockwave [7], heavy-slow resistance training [8, 9] and corticosteroid injections [10, 11], patients may experience symptoms for several years [12].

The usefulness of ultrasonography for the diagnosis of plantar fasciopathy is widely established [13, 14]. Ultrasonographic findings in patients with plantar fasciopathy include hypoechogenicity and a thickness of the plantar fascia of 4 mm or more [14]. Despite the relevance of ultrasonography in plantar fasciopathy, the measurement characteristics such as the positioning of the patient, the positioning of the metatarsophalangeal joint and probe (sagittal versus frontal plane) have been mostly ignored and are not necessarily reported in studies, which hampers replicability [14]. The European Society of Musculoskeletal Radiology [15] recommends that the patient is in a prone position with the toes resting on the examination table to keep the foot perpendicular to the leg but most studies use a position where the feet are hanging freely over the end [14]. Granado et al. recently found that different positions of the metatarsophalangeal joints influenced the thickness of the plantar fascia when the feet were hanging freely over the end of the table [16]. This indicates that it is of importance to further investigate the effect of patient positioning.

Other examinations performed on the plantar fascia may also be affected by whether the plantar fascia is stretched or not. Pressure pain thresholds are a measure of pain sensitivity and are usually investigated by applying pressure on the skin with a pressure algometer until the patient first experiences pain [17]. Past studies of pressure pain thresholds in patients with plantar fasciopathy compared to pain-free controls have found conflicting evidence; three studies found that patients had lower pressure pain thresholds [18–20] whereas one did not find any differences [21]. This could potentially be due to different metatarsophalangeal joint angles during testing, however, only two in four studies reported the positioning of the patient during testing [18–21]. To allow for replication in future studies, it is important to know if stretching the plantar fascia affects the pressure pain threshold.

The primary aim of this study was to compare the thickness of the plantar fascia in stretched and relaxed positions in patients with plantar fasciopathy. We hypothesised that the plantar fascia would be thinner in the stretched position. The secondary aims were to compare the plantar fascia thickness in the sagittal and frontal planes, and to compare the pressure pain thresholds at the most tender spot under the plantar heel using pressure algometry in both a stretched and relaxed plantar fascia and in the most and least symptomatic foot. We hypothesised that the thickness would be the same in the sagittal and frontal planes and that the pressure pain threshold would be lower in the stretched position and in the most symptomatic foot. We also hypothesised that a thicker plantar fascia would have a lower pressure pain threshold and, thus, we wanted to investigate any association between these two measures.

## Methods

### Study design and recruitment

This study was conducted as an observational cross-sectional in patients with plantar fasciopathy conducted in the municipality of Aalborg. The reporting of the study follows the STrengthening the Reporting of OBservational studies in Epidemiology (STROBE) Statement. Patients were recruited from local general practice and rheumatology clinics in Denmark between September 2017 and April 2018.

### Eligibility criteria

The criteria for participation were in line with those of similar studies in this patient population [9, 11, 22]. Diagnosis of plantar fasciopathy was made based on patient history and clinical examination as follows: 1. self-reported heel pain of at least 3 months duration: 2. pain and/tenderness on palpation of the medial tubercle of the calcaneus 3. plantar fascia thickness of 4 mm or greater, as measured by ultrasound examination. In addition, participants were required to have a pain intensity (average heel pain in the past week) of a minimum of 3 on a numeric pain rating scale (NRS) ranging from 0 (no pain) to 10 (worst heel pain imaginable). Exclusion criteria were less than 18 years of age; history of systemic disease; pain medication in the last 24 h; steroid injection in the previous 6 months; previous heel surgery or fracture of the lower leg or foot. Diagnosis and inclusion were undertaken by SO, medical doctor and authorised resident in general practice who received training in the study procedures by JLO, an experienced rheumatologist, and by HR, an experienced physiotherapist, who have several years of experience with ultrasonography and pressure algometry. Furthermore, a pilot study of four patients was conducted by SO before the inclusion of the first study participant.

### Procedure

Participants were required to attend a single testing session, which consisted of diagnosis and inclusion, self-report questionnaires (consisting of demographic data, including sex, age, height, weight, pain duration, whether participants had unilateral or bilateral pain, and if bilateral, the self-evaluated most painful limb), followed by measurement of plantar fascia thickness (relaxed and stretched), and pressure pain thresholds. Measurements were taken first on the symptomatic (or most symptomatic foot in cases of bilateral pain), followed by the contra-lateral foot.

### Ultrasound scanning

**Fig. 1:**
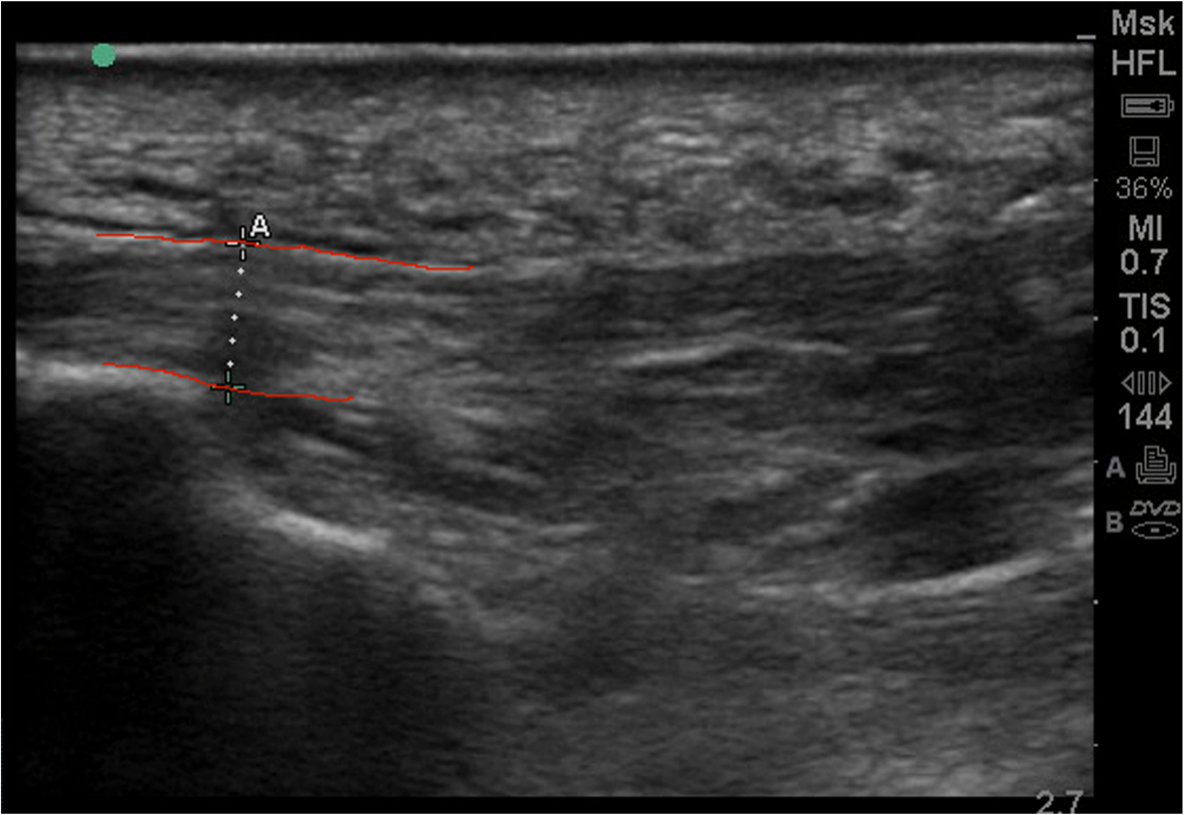
Image of measurement in sagittal plane. The borders of the plantar fascia are highlighted in red

**Fig. 2:**
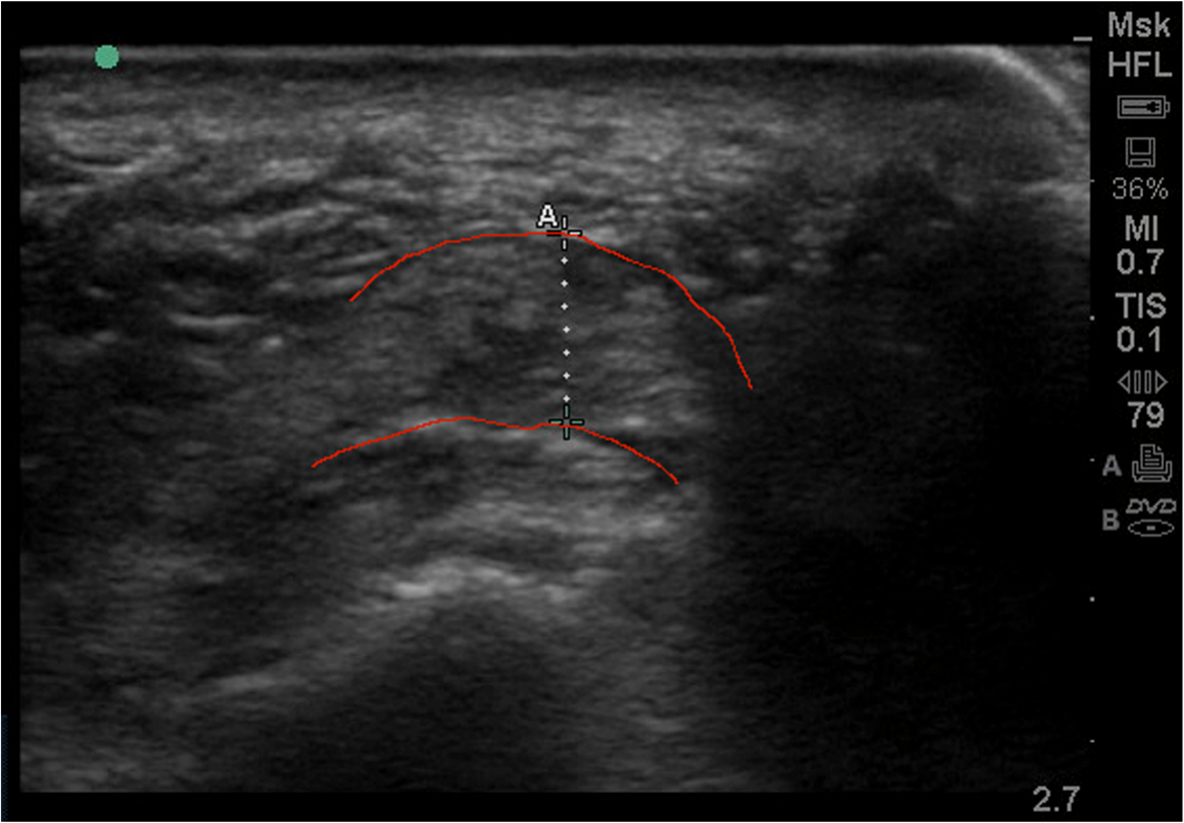
Image of measurement in frontal plane. The borders of the plantar fascia are highlighted in red

To measure plantar fascia thickness, participants were positioned in a prone position on an examination bed, with their feet hanging over the end of the bed in a relaxed manner. Plantar fascia ultrasound scans were taken using the SonoSite M-Turbo® with a linear transducer (6–13 MHz transducer frequency). The transducer was placed on the plantar surface of the heel in the sagittal plane aiming towards the second toe. Scans were taken at the attachment of the plantar fascia to the medial calcaneal tuberosity. The thickness was measured by manually selecting the two points perpendicular to the plantar fascia at the insertion on the calcaneus and directly measuring the distance in the ultrasound software (see Figs. 1 and 2). This procedure was repeated three times on three different applications of the probe and the average was used for analyses. This has been found to be a reliable method (ICC = 0.67 to 0.77) [23]. Subsequently, the thickness was measured in frontal plane, again three times. The transducer was placed in the frontal plane over the plantar heel. Measurements were first conducted on the (most) symptomatic foot, followed by the contra-lateral foot.

To measure the plantar fascia thickness in a stretched position, the participant was lying in prone with the knee fully extended and toes maximally dorsi-flexed on the examination table. In this position, the measurement of the plantar fascia was repeated as previously outlined.

### Pressure pain thresholds

Pressure pain thresholds were taken with participants lying in prone, with their feet hanging over the end of the table in a relaxed position. The most painful point on the heel was determined by palpation and marked [20, 22]. A hand-held algometer (Somedic, Hörby, Sweden) with a 1-cm2 probe was then placed perpendicular to the skin at the marked site. The assessor increased the pressure at a constant rate of 30 kPa/s [21, 22, 24–26]. Participants were instructed to press a handheld switch the first instance they felt the sensation change from pressure only, to pain. The pressure at this point was recorded as the pressure pain threshold. This procedure was repeated three times, with a 30-s interval between tests. The average of these three tests was used for analyses [20, 21, 24, 27]. The process was then repeated on the least symptomatic foot. In cases of unilateral pain, the pressure pain threshold was taken at a standardised location on the anteromedial aspect of the heel [20].

To measure the pressure pain threshold with the plantar fascia in a stretched position, the above procedure was repeated with the participant lying in prone with the knee fully extended and toes maximally dorsi-flexed on the examination table. Again, this was done bilaterally.

### Sample size

The sample size was estimated based on data from Wen-Chung Tsai and colleagues [28]. At the time of sample size calculation, no data on different testing positions were available. Therefore, we used previous measurements of the plantar fascia thickness in patients with plantar fasciopathy comparing most and least affected limbs. Using an estimated fascia thickness of 5.61 mm (±1.19) in the relaxed position and a mean thickness of 4.86 mm (±1.0) in the stretched position, it was estimated that with an alpha of 0.05 and power of 0.8, a sample of 20 participants would be needed. Sample size calculation was undertaken in G*power 3.1.

### Statistical analyses

SPSS (IBM Corporation, New York, United States) was used for statistical analyses. Normal distribution was visually assessed from histograms. To compare thickness and pressure pain thresholds in stretched and relaxed positions of the plantar fascia on most and least symptomatic sides paired-t-tests were applied. The association between the thickness of the plantar fascia and pressure pain threshold was investigated using the Pearson correlation coefficient.

## Results

### Participants

**Fig. 3:**
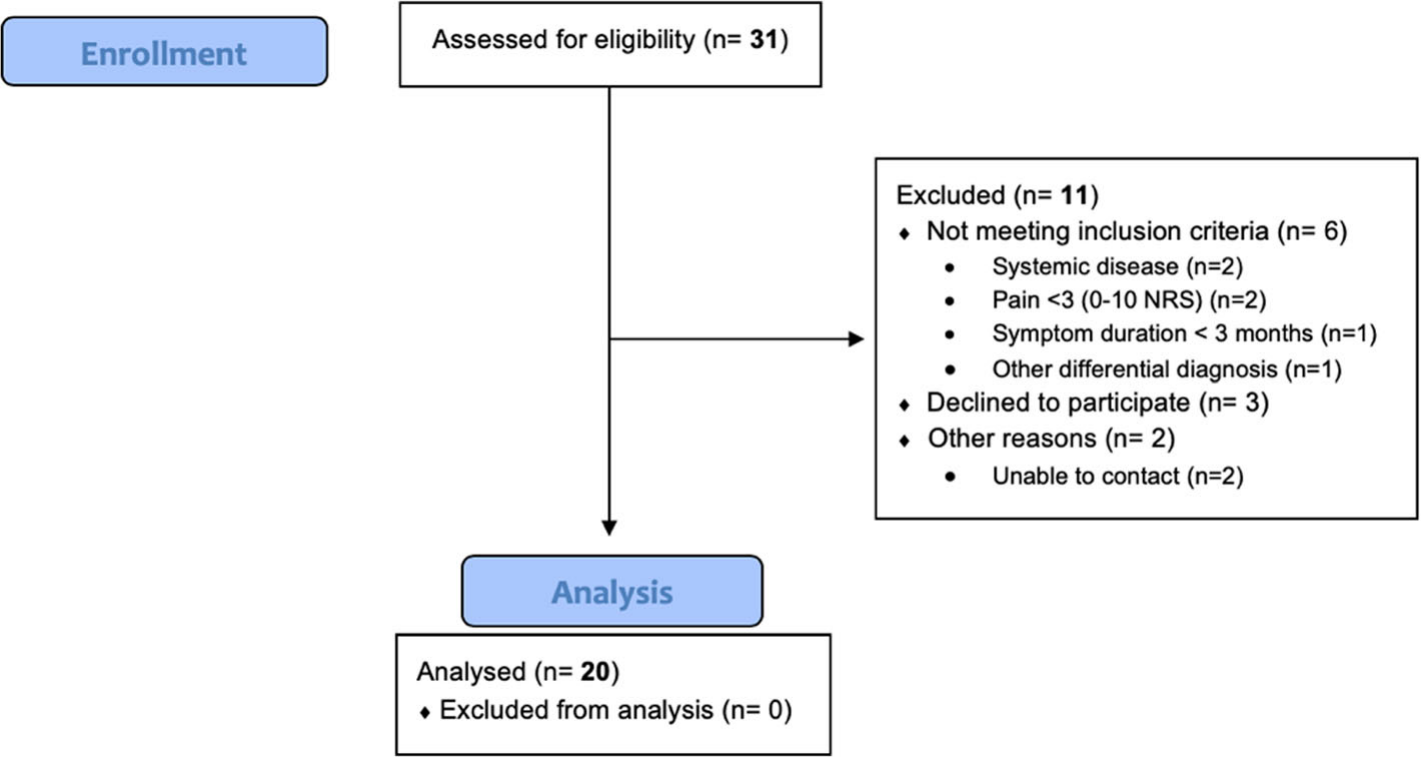
Flowchart of recruitment

After having screened 31 potential participants, we included 20 individuals with plantar fasciopathy (see flow chart, Fig. 3). They were predominantly female (18/20), had a mean age of 52 (±11) years and a BMI of 30.7 (±5.9). Participants had an average heel pain intensity of 5.4 (±2.1) during the week prior to the examination and they had had symptoms for a median of 11.5 (IQR 5.5–19.5) months. The majority had only unilateral pain (13/20). All experienced first step pain in the morning, 12 experienced pain relief during the day and all but one participant felt an exacerbation of symptoms in the evening or after participating in physical activities.

#### Outcome measures

**Fig. 4:**
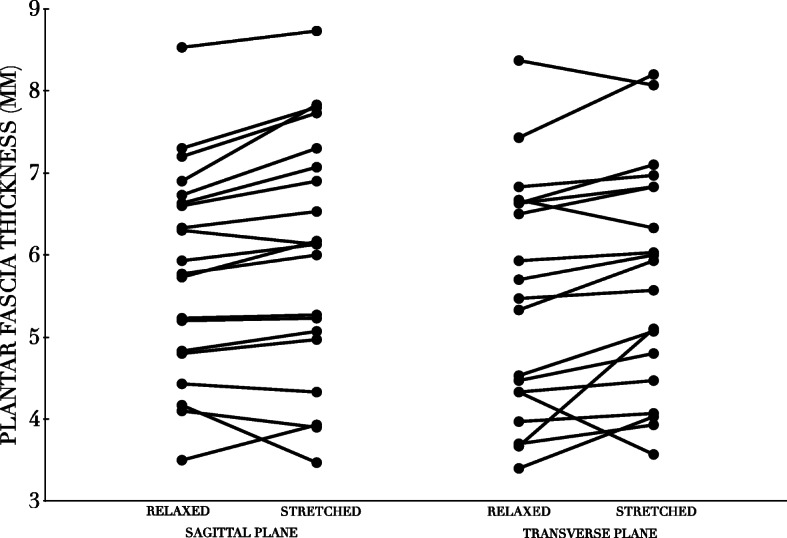
Individual measurements using ultrasonography of the most symptomatic limb

We found a significantly thicker plantar fascia for the most symptomatic foot measured in the sagittal plane when it was stretched compared with the relaxed position (mean difference 0.2 mm, 95%CI: 0.1–0.4, *P* = 0.013) and a thicker plantar fascia measured in the sagittal plane versus the frontal plane (Tables 1 and 2, Fig. 4). There was no difference between pressure pain thresholds in stretched or relaxed positions in either foot (Table 2). The pressure pain threshold was higher in the least symptomatic foot compared with the most symptomatic foot in both stretched and relaxed positions (*P* < 0.001 and *P* = 0.008, respectively). There was no correlation between pressure pain thresholds and the plantar fascia thickness in the stretched position (*r* = 0.15, *P* = 0.054) nor in the relaxed position (*r* = − 0.18, *P* = 0.444).

**Table 1:** Plantar fascia thicknesses and pressure pain thresholds

**PLANTAR FASCIA THICKNESS** **mm (SD)**		
	**STRETCHED**	**RELAXED**
**MOST SYMPTOMATIC**		
**SAGITTAL**	6.0 (1.5)	5.8 (1.2)
**FRONTAL**	5.8 (1.4)	5.5 (1.4)
**LEAST SYMPTOMATIC**		
**SAGITTAL**	4.6 (0.8)	4.6 (0.9)
**FRONTAL**	4.6 (1.0)	4.4 (1.0)
**PRESSURE PAIN THRESHOLDS** **kPa (SD)**		
	**MOST SYMPTOMATIC**	**LEAST SYMPTOMATIC**
**STRETCHED**	401.6 (101.7)	503.7 (155.4)
**RELAXED**	388.1 (110.5)	492.7 (159.0)

**Table 2:** Results of analyses

	MEAN DIFFERENCE (95% CI)	***P***-VALUE
**PLANTAR FASCIA THICKNESS (mm)**		
**STRETCHED VS RELAXED SAGITTAL**	−0.21 (− 0.38 to − 0.05)	0.013
**STRETCHED VS RELAXED FRONTAL**	−0.27 (− 0.49 to − 0.06)	0.014
**FRONTAL VS SAGITTAL STRETCHED**	− 0.23 (− 0.42 to − 0.03)	0.025
**FRONTAL VS SAGITTAL RELAXED**	−0.29 (− 0.49 to − 0.08)	0.008
**MOST VS LEAST SYMPTOMATIC SAGITTAL**	1.39 (0.72 to 2.07)	< 0.001
**MOST VS LEAST SYMPTOMATIC FRONTAL**	1.25 (0.47 to 2.02)	0.003
**PRESSURE PAIN THRESHOLDS (kPa)**		
**STRETCHED VS RELAXED MOST SYMPTOMATIC**	−13.5 (−47.5 to 20.4)	0.414
**MOST VS LEAST SYMPTOMATIC RELAXED**	− 104.7 (− 177.9 to −31.4)	0.008
**STRETCHED VS RELAXED LEAST SYMPTOMATIC**	−10.9 (−61.6 to 39.8)	0.657
**MOST VS LEAST SYMPTOMATIC STRETCHED**	− 102.1 (− 150.4 to − 53.7)	< 0.001

## Discussion

In this cross-sectional study, we investigated whether the ultrasound-measured thickness of the plantar fascia and pressure pain threshold would be affected by stretching the plantar fascia. We found that the plantar fascia was significantly thicker in a stretched compared with a relaxed position. There was no difference in pressure pain threshold between the two positions.

Contrary to our hypothesis and to the findings by Granado et al. [16], we found that the plantar fascia was significantly thicker in the stretched position. Granado et al. found a difference of 0.4 mm, whereas we found a difference of 0.2 mm. Both studies used similar methods in terms of the equipment and placement of the measurement markers. However, the definition of maximal dorsiflexion of the toes was different. Granado et al. asked patients to actively dorsi-flex the metatarsophalangeal joints maximally whereas we asked patients to place their toes against the examination table which caused a maximal passive plantar fascia stretch. The passive range of motion of the metatarsophalangeal joints in patients with plantar fasciopathy is greater than the active range of motion, thus, it is likely that the participants of our study dorsi-flexed the toes more than those of Granado and colleagues [29]. However, this does not explain why we found that a stretched plantar fascia was thicker than a relaxed plantar fascia as we hypothesised that stretching the fascia would decrease the thickness. The basis for that hypothesis was the viscoelastic properties of tendons which may also be associated with a decreasing thickness after resistance exercise [30]. Therefore, stretching the fascia could have led to similar changes in thickness. A feasible explanation may be that the measurement differences between the positions were caused by differences in the ease of identifying the fascia on the images rather than structural changes of the fascia. It has previously been suggested that a delineation of the surface of the fascia is easier when the fascia is stretched which makes it less challenging to place the markers for measuring [28]. Yet, Granado et al. should have found the same despite using a position with less dorsiflexion. We did only find a difference of 0.2 mm between the two positions which is less than the standard deviation of measurements of asymptomatic fasciae and the clinical importance of such a small difference is questionable [23]. However, the reason for variation in measurements of the symptomatic fascia remains unknown and should be studied in the future. Based on our results as well as those of Granado et al., the positioning of the metatarsophalangeal joints is associated with a change in measurements of the plantar fascia which is why the position used in research should be reported. Furthermore, when using repeated measurements over time, the reliability of doing so would increase if the same position is used consistently.

In both the most and least symptomatic limb, we found a thinner plantar fascia in the frontal versus sagittal plane. Hence, changing the plane in which ultrasonography is being performed may be associated with conflicting measurements of the plantar fascia and the plane should be reported in research and clinicians should be consistent with the method applied in the clinic. The difference between measurements in these two planes may be caused by angulation. The transducer may be tilted medially and laterally when performing a scan in the sagittal plane, whereas the transducer may also be tilted in a proximal and distal angle when a scan in the frontal plane is performed. This has been suggested to affect measurement error when performing ultrasonography of both the Achilles and patellar tendons [31]. The measurement point when performing a scan in the frontal plane may be slightly more distal than in the sagittal plane. This could explain why we found a thinner fascia in this plane as the region of maximal thickness is usually near the insertion on the calcaneus. It may be easier to identify the insertion on the calcaneus in the sagittal plane compared to the frontal plane as the calcaneus is shown in the sagittal image whereas, when in the frontal plane, the transducer has to be moved distal to the calcaneus to place the measurement markers. Thus, it is likely that the measurement is made 1 or 2 mm more distal to the calcaneus compared to the measurement in the sagittal plane. We did not aim to investigate inter-subject reliability of the performing measurements in the frontal plane, but this may be of relevance in the future.

A cardinal feature of plantar fasciopathy is pain when pressure is applied at the insertion of the plantar fascia on the calcaneus [32]. Despite this, there has been conflicting evidence of decreased pressure pain thresholds as three studies found that patients had lower pressure pain thresholds whereas one did not find any differences when patients with plantar fasciopathy were compared with pain-free controls [18–21]. We found that the pressure pain threshold was significantly lower in the most symptomatic limb compared to the least symptomatic limb. This suggests that patients with plantar fasciopathy experience an increased local pain sensitivity. We did not find that pressure pain thresholds were affected by changing the metatarsophalangeal joint position. Though we did not power the study to find a difference in pressure pain thresholds, it appears unlikely that a difference exists based on our findings. Therefore, both methods could be of interest should they be implemented in clinical practice. Plantar fasciopathy is associated with pain when the great toe is dorsal-flexed due to the windlass mechanism, thus, the relaxed position may be preferred by patients [33].

We hypothesised that there would be an association between plantar fascia thickness and pressure pain thresholds as larger increases in fascia thickness could have indicated a larger severity of the condition. We found no such association which is in line with previous research demonstrating that the plantar fascia thickness in patients with plantar fasciopathy is not associated with either self-reported pain nor function [34]. Measuring the plantar fascia thickness may, therefore, only be relevant to support diagnosing the condition.

### Strengths

We used a single assessor who performed all measurements throughout the study. Rather than relying on a single measurement, we used an average of three measurements both during ultrasonography and measurements of pressure pain thresholds which increases the reliability [23].

### Limitations

One limitation is that the assessor was not blinded to the ultrasonographic measurements. Because we used pressure pain thresholds at the most tender point rather than a standardised location, it was not possible to blind the pressure pain threshold assessments. The use of a goniometer would have allowed us to monitor the metatarsophalangeal joint position as it could have varied if participants did not keep the knee constantly extended.

## Conclusions

The plantar fascia was significantly thicker in a stretched compared with a relaxed position and in the sagittal compared with the frontal plane, but differences were smaller than the standard deviation. Pressure pain thresholds were not affected by the different positions. These results highlight the importance of how ultrasonography is performed and reported in research to allow for replication.

## Data Availability

Data is available upon reasonable request.

## Abbreviations

ICC: Intraclass correlation coefficient
IQR: Interquartile range
